# Vaccine Strategies for the Control and Prevention of Japanese Encephalitis in Mainland China, 1951–2011

**DOI:** 10.1371/journal.pntd.0003015

**Published:** 2014-08-14

**Authors:** Xiaoyan Gao, Xiaolong Li, Minghua Li, Shihong Fu, Huanyu Wang, Zhi Lu, Yuxi Cao, Ying He, Wuyang Zhu, Tingting Zhang, Ernest A. Gould, Guodong Liang

**Affiliations:** 1 State Key Laboratory for Infectious Disease Prevention and Control, National Institute for Viral Disease Control and Prevention, Chinese Center for Disease Control and Prevention, Beijing, People's Republic of China; 2 Collaborative Innovation Center for Diagnosis and Treatment of Infectious Diseases, Hangzhou, People's Republic of China; 3 Unité des Virus Emergents, Aix-Marseille University Faculté de Médecine de Marseille, Marseille, France; Emory University, United States of America

## Abstract

Japanese encephalitis (JE) is arguably one of the most serious viral encephalitis diseases worldwide. China has a long history of high prevalence of Japanese encephalitis, with thousands of cases reported annually and incidence rates often exceeding 15/100,000. In global terms, the scale of outbreaks and high incidence of these pandemics has almost been unique, placing a heavy burden on the Chinese health authorities. However, the introduction of vaccines, developed in China, combined with an intensive vaccination program initiated during the 1970s, as well as other public health interventions, has dramatically decreased the incidence from 20.92/100,000 in 1971, to 0.12/100,000 in 2011. Moreover, in less readily accessible areas of China, changes to agricultural practices designed to reduce chances of mosquito bites as well as mosquito population densities have also been proven effective in reducing local JE incidence. This unprecedented public health achievement has saved many lives and provided valuable experience that could be directly applicable to the control of vector-borne diseases around the world. Here, we review and discuss strategies for promotion and expansion of vaccination programs to reduce the incidence of JE even further, for the benefit of health authorities throughout Asia and, potentially, worldwide.

## JE in Mainland China

**Figure 1:**
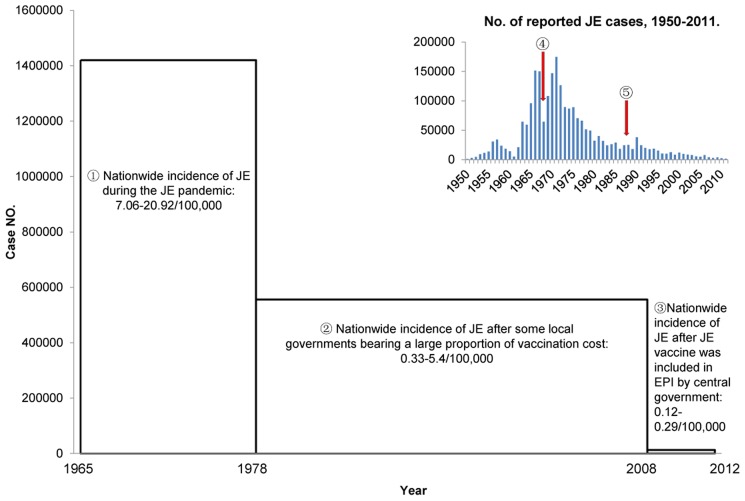
The role of immunization in JE control and prevention in China. The thumbnail in the upper right corner is cited from reference [1]. The bar indicates JE case numbers. 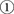 The JE pandemic in China peaked during 1965–1977. 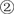 JE incidence decreased significantly after the local government covered a large proportion of immunization costs. 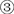 In 2008, the JE vaccine was included in the EPI by the central government, and JE immunization became free throughout China. Subsequently, JE has been controlled and the incidence remains at a low level. 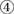 The arrow indicates the introduction of the P3 inactivated JE vaccine in 1968. 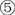 The arrow indicates the point at which the SA 14-14-2 live-attenuated JE vaccine was licensed for use in China in 1988.

**Figure 2:**
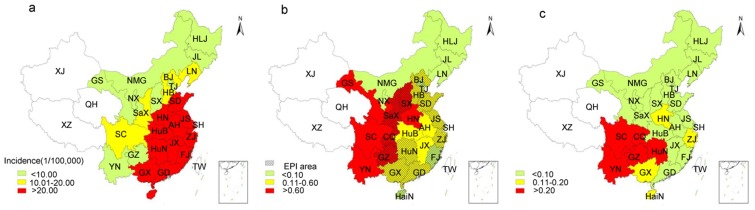
Incidence of JE in China. (a) In 1971, during the JE pandemic, the 11 provinces where JE incidence was higher than the national average (20.92/100,000) were mainly distributed in the coastal areas of eastern China. The colors red, yellow, and light green in panel “a” indicate provinces with incidence higher than 20.00/100,000, 10.01–20/100,000 and lower than 10.00/100,000, respectively. (b) In 2006, the necessary local governments provided a large proportion of immunization costs, and JE incidence rates in economically developed regions of eastern China were far lower than the national average (0.58/100,000). The colors red, yellow, and light green in panel “b” indicate provinces with incidence higher than 0.60/100,000, 0.11–0.60/100,000, and lower than 0.10/100,000, respectively. The grid shadow showed 16 provinces that had implemented JE immunization programs by 2006. (c) In 2011, after the JE vaccine was included in the EPI, Sichuan, Yunnan, Guizhou, and other underdeveloped areas in southwest China still had JE incidence rates higher than the national average (0.12/100,000). The colors red, yellow, and light green in panel “c” indicate provinces with incidence higher than 0.20/100,000, 0.11–0.20/100,000 and lower than 0.10/100,000, respectively. The blank areas in panels “a,” “b,” and “c” represent Xinjiang, Tibet, and Qinghai, respectively, which are considered JE-free areas. The data of JE cases in Taiwan (blank) are not included in the statistics. **Abbreviations:** HLJ: Heilongjiang; JL: Jilin; LN: Liaoning; NMG: Inner Mongolia; BJ: Beijing; TJ: Tianjin; HB: Hebei: HN: Henan; SD: Shandong; SX: Shanxi; SaX: Shaanxi; NX: Ningxia; GS: Gansu; QH: Qinghai; XJ: Xinjiang; XZ: Tibet; SC: Sichuan; CQ: Chongqing; YN: Yunnan; GZ: Guizhou; GX: Guangxi; GD: Guangdong; GX: Guangxi; HaiN: Hainan; HuN: Hunan; HuB: Hubei; JX: Jiangxi; JS: Jiangsu; ZJ: Zhejiang; AH: Anhui; FJ: Fujian; SH: Shanghai; TW: Taiwan.

JE is highly endemic in China with thousands of cases reported annually [1], [2]. The first clinical case of JE was confirmed in 1943, and Japanese encephalitis virus (JEV) was subsequently isolated in 1949 [1]. Since then, more cases were found and many viruses were isolated [1], [3]. As JE was epidemic in most regions of mainland China and caused a huge disease burden, it was classified as a National Notifiable Infectious Disease in 1950 [1], [2]. More than 2 million cases were reported in China between 1950 and 2011 (Figure 1) [1], [2], [4]–[31]. The number of cases reached a peak during the 1960s–1970s, when no vaccine was available. Furthermore, China was in the period of the “Cultural Revolution” (1966–1976), which resulted in a major decline of the national economy and extremely low investment in public health. As a result, mainland China experienced a natural state of JE pandemics during the 1960s and 1970s in the absence of vaccines and other public health interventions (Figure 1). From 1965 to 1977, a total of 1.4 million JE cases, accounting for more than 50% of the total number during the period from 1950 to 2011, were reported in 26 of China's 29 provinces (incidence: 7.06–20.09/100,000) [4]. Taking 1971 as an example (Figure 2a), there were 11 provinces where the incidence of JE was higher than 20/100,000 [32]–[42]. These provinces were Jiangsu, Shandong, Zhejiang, Guangdong, Guangxi, Anhui, Henan, Hubei, Hunan, Jiangxi, and Fujian, most of which are located in eastern coastal areas. Subsequently, with the continuous promotion of immunization using Chinese vaccines, the incidence and number of cases of JE has gradually decreased (Figure 1).

## Role of Vaccine in JE control

### JE vaccine in mainland China

JE is a focal disease caused by JEV, which circulates in natural habitats between mosquitoes and vertebrate hosts, such as birds, bats, and wild pigs, as well as domestic pigs [43]. Humans are incidental hosts and their immunization will not impact the virus in these natural habitats. Therefore, by analogy with other viruses, such as yellow fever virus in the jungles of Africa and South America, JE is almost impossible to eliminate. Nevertheless, immunization has proven to be the optimal procedure to protect healthy people from JEV infection in China. Consequently, following the isolation of JEV in China, a great deal of research was conducted to develop a suitable JE vaccine and accordingly, both inactivated and live-attenuated JE vaccines were successfully developed in the hope of resolving the public health problem of JE morbidity and mortality in China.

The first vaccine, the formaldehyde-inactivated JEV (P3 strain) derived vaccine, was developed in China. It was derived from laboratory-infected suckling mouse brains and was successfully developed, safety tested, and manufactured in 1968. Improved versions followed, firstly by the development of primary hamster kidney cell–derived inactivated JE vaccine, and secondly, by the Vero cell–derived inactivated JE vaccine [44], [45]. From 1968 on, the P3 inactivated JE vaccine began to be used; however, it was limited to certain small areas because of low production capacity. A total of five doses was administered, two at 12 months of age with a one week interval, followed by three doses at ages 2, 6, and 10 years old. Only the inactivated P3 vaccine was used until 1988. Subsequently in 1988, the first live-attenuated vaccine (using the SA14-14-2 strain) was licensed for use in China [45]–[47]. Individuals were immunized with two doses of the SA 14-14-2 live-attenuated JE vaccine, at 8 months and then 2 years later [48]. In the long term, both the P3 inactivated JE vaccine and the SA 14-14-2 live-attenuated JE vaccine were used. Compared with inactivated vaccines, the live-attenuated vaccine was less expensive to produce and administer; it was shown to be safe for use in both young and elderly individuals, required fewer doses per individual, and the immunization procedure was easier to apply [49]. Therefore, the use of the SA 14-14-2 live-attenuated JE vaccine gradually increased and eventually replaced the P3 inactivated JE vaccine. Since 2008, the live-attenuated vaccine has accounted for almost 100% of JE vaccines administered in China after its inclusion in the national Expanded Program of Immunization (EPI). Four companies produce this type of vaccine in mainland China (Chengdu Institute of Biological Products, Wuhan Institute of Biological Products, Lanzhou Institute of Biological Products, and Shanghai Institute of Biological products). More than 300 million doses of live-attenuated JE vaccine were produced from 2007–2011 to meet not only domestic demands, but overseas markets, such as Nepal and India [50].

In addition to the P3 inactivated JE vaccine and SA 14-14-2 live-attenuated JE vaccine, there are other JE vaccines that are used worldwide. The inactivated mouse brain–derived Nakayama JE vaccine and Beijing-1 JE vaccine were produced and used in Japan, India, South Korea, Taiwan, Thailand, Vietnam, and elsewhere. However, since the end of 2005, the production of these two inactivated mouse brain–derived vaccines has ceased. The Vero cell–derived Beijing-1 JE inactivated vaccine (JEBIK V) made by Biken had been available since 2009 [48]. An inactivated vaccine based on the SA 14-14-2 attenuated virus made by Intercell (IXIARO), used to immunize adults (17 years of age and older) in Europe, Hong Kong, Australia, Canada, and the US, was also approved for pediatric use during 2013 (http://www.ncbi.nlm.nih.gov/pmc/articles/PMC3486421/).

### JE immunization

Although the inactivated JE vaccine was first developed in 1968, the Chinese government was unable to cover the costs of immunization throughout China because of the underdeveloped economy at that time. Therefore, immunization remained the privilege of only individuals who could cover all costs (including the vaccine, syringe, and needle). At this time of economic frailty, most Chinese people, especially those in rural areas with large families, were unable to afford the costs of immunization. In addition, vaccine production was low because of limited production capacity. Hence, immunization coverage with the vaccine was low and had no significant impact on the JE pandemic during 1971 to 1972. However, following improvements in the national economy, China implemented an improved JE vaccination program. Initially, the government provided a high proportion of the immunization costs, with individuals contributing a small proportion towards the costs. Subsequently, as the economy continued to improve, the EPI was introduced, which provided full costs and free immunization. These measures have greatly improved JE immunization coverage, and the risk of infection by JEV in mainland China has been reduced to very low levels, as will be described in more detail below.

### 1. First phase of JE immunization: The government pays a large part of the costs and individuals pay a small proportion

As individuals had to cover the cost of immunization, the coverage level was low. At this early time, JE immunization was carried out predominantly amongst families on the higher socioeconomic scale in large and medium-sized cities, but rarely in rural regions where the incidence of JE was high. However, with China's reform and economic recovery, particularly in eastern coastal regions, local governments were able to increase investment in public health, and priority funds were allocated to JE immunization of school-aged children. For example, JE was highly endemic in the Jiangsu province during the pandemic period of the 1970s. Around 30,000 JE cases were reported in 1971 (incidence: 53.06/100000), which was the highest among all provinces [32]. However, since 1978, a JE vaccination program in which the government had paid a high proportion of the costs for school-aged children, had been continuously implemented in the Jiangsu province. At that time, under the funds provided by local government, the public health department was responsible for the purchase of the JE vaccine, cold-chain transportation, storage, and other related public outreach costs. The local government contributed 14 renminbi (RMB) per dose of JE vaccine, leaving individuals to cover only 1 RMB for the syringe. This resulted in most rural families accepting the small cost of immunization, and a significant improvement in JE immunization coverage was recorded in Jiangsu, resulting in 97.3% coverage for primary immunization and 92.7% coverage for the booster inoculation in 2005. Correspondingly, the incidence of JE in Jiangsu decreased to 0.09/100,000 in 2005, with only 66 reported cases of JE [32]. In conclusion, the strategy of high vaccination coverage was shown to play a key role in local JE prevention and control.

Several other economically developed provinces also began to provide partially free immunization programs (mainly paid for by the government, with individuals covering only the cost of the syringe) for school-aged children. Thirteen provinces, viz., Beijing, Tianjin, Shanghai, Jiangsu, Zhejiang, Fujian, Shandong, Hainan, Hebei, Shaanxi, Hunan, Chongqing, and Guangdong, had implemented such JE immunization programs by 2005 [32], [33], [35], [40], [42], [51]–[58]. Another three provinces, including Guizhou, Guangxi, and Shanxi subsequently joined in [59]–[61], and by 2006, 16 provinces were funding the immunization of local children. Thus, as the government was able to provide increasing funding for JE immunization for children, coverage clearly increased, and the incidence of JE was markedly reduced, even in rural areas of the central-eastern provinces, i.e., areas that had previously experienced high JE incidence rates during the pandemic period. During the 5 years from 2003 to 2007 in which JE immunization was paid for mainly by the government, the nationwide JE incidence rate was 0.33–0.58/100,000, which was 30–40 times lower than that recorded in the 5-year period from 1969 to 1974 during the pandemic period (9.67–20.92/100,000) (Figure 1) [1], [2]. Compared with the pre-JE vaccine era, taking 1971 as an example (Figure 2a), the JE incidence rate in the central-eastern provinces dropped below the national average (0.5/100,000) as recorded in 2006, due to the use of vaccine in these areas (Figure 2b) [62]. As JE incidence in these provinces fell to a low level, the nationwide incidence also decreased. Therefore, the government-funded JE immunization programs significantly reduced the incidence of the disease.

### 2. Second phase JE immunization: Inclusion in the EPI

Although the incidence of JE decreased markedly in some provinces following introduction of government-funded immunization, the nationwide immunization coverage was still low. As a result, the morbidity and mortality of JE remained within the top five of the National Notifiable Infectious Diseases between 1980 and 1995 [63]. However, with the increase of gross domestic product (GDP), the marked economic improvement enabled the government to expand investment in public health (Figure 1). In 2007, the central government announced its intention to include the development of vaccines against 15 infectious diseases (including JE) in the national EPI, implying that the government would bear the full cost of JE immunization [1], [2], [64]. Since 2008, 28 provinces (municipalities or autonomous regions) have implemented cost-free JE immunization, with the exception of areas in which JE is not prevalent, such as Xinjiang, Tibet, and Qinghai. Moreover, the central government has allocated special funds to help underdeveloped southwestern areas (such as Guizhou and Yunnan) implement the immunization program to alleviate their economic burden. Consequently, national JE immunization coverage has increased significantly, and the incidence of JE has decreased further. Vaccines have played an increasing role in JE prevention and control since 2008 (Figure 1) [29]–[31], [65]. The number of reported cases was markedly reduced from around 30,000 (incidence: 0.33–0.58/100,000) in the 5-year period from 2003 to 2007, when only a partial immunization program was implemented, to about 13,000 (0.12–0.29/100,000) in the 5-year period from 2008 to 2012, when the JE vaccine was included in the EPI [23]–[31], [65].

### Economic and social benefits of JE immunization

Inclusion of the JE vaccine in the EPI not only decreased the number of reported JE cases, but also yielded a number of economic and social benefits. A cohort study using a cost-effectiveness model was conducted to assess the economic and social benefits of JE immunization in Guizhou, located in southwest China [66]. In this study, researchers modeled hypothetical cohorts of 100,000 people recorded over a period of 65 years, and the results indicated that spending $400,000 to immunize 100,000 individuals could prevent 406 JE cases, 102 deaths, and 122 cases of chronic disability (equivalent to 4,554 disability adjusted life years—DALYs). Taking into account the costs required for future treatment, immunization of 100,000 people could save about $1.6 million for the health budget and $11 million from the societal perspective. Therefore, integrating the JE vaccine into the EPI is a cost-effective investment. Interestingly, a similar study in Shanghai [67] indicated that, under the assumption of a 30-year follow-up, the average direct cost of a single case of JE would be $1,512 (calculated with the exchange rates in 1997), excluding indirect costs. Immunizing 100,000 people with the inactivated vaccine or the live-attenuated vaccine could therefore save $348,246 and $512,456, respectively (considering only the direct costs of treatment). Taking the series of high, indirect costs in large cities into consideration, JE immunization conducted in urban areas would have significant cost benefits. In other words, promoting JE immunization programs on a national scale, in both urban and rural areas, would significantly reduce public health costs.

## Impact of Economic and Social Factors on JE Prevention and Control

In addition to vaccination, economic and social factors also play important roles in JE prevention and control. Economic development can enable local governments not only to integrate JE vaccination into the EPI but also to implement comprehensive management for JE control, such as improving the public health environment, for example, by moving pig farms away from villages, improving sewage disposal to reduce mosquito breeding, and implementing mosquito control plans during the mosquito breeding season [68]. Economic development and improved living standards can also positively influence JE prevention and control. For example, as villagers move from cottages close to pigsties and paddy fields into buildings far from livestock and farmland, the chance of being bitten by a mosquito is reduced. In addition, farming methods and patterns are changing. Some areas that were traditionally dominated by crop planting have changed to growing commercial crops, such as fruit trees, which require less irrigation, and thereby reduce the level of mosquito breeding. These changes in farming practices have also impacted the indigenous mosquito species due to changes in the ecological environment; *Culex tritaeniorhynchus*, the major transmission vector of JEV, was the dominant species in previous years, but is now rarely found in areas such as Inner Mongolia [69]. The measures described above have also reduced the level of natural circulation of JEV between mosquitoes and pigs and thus have decreased the risk of JEV infection amongst the human population.

After inclusion of JE immunization in the EPI in 2008, the national JE incidence rate remained at a low level. However, the regions with relatively higher JE incidence rates switched from eastern coastal areas to the underdeveloped southwest regions, such as Yunnan, Guizhou, and Sichuan because of the unbalanced economic development in these regions. For example, the national JE incidence was 0.12/100,000 in 2011 [31], while that of these southwest regions was greater than 0.2/100,000, which was far higher than the national average (Figure 2c). Yunnan, Guizhou, and Sichuan are located in southwest China, which is mainly surrounded by mountains, with few arable areas, and the houses, pigsties, and paddy fields (rice is the main local crop) are located in a narrow strip of arable land. In this type of special microenvironment, mosquitoes breed in paddy fields, and thus JEV spreads readily, causing human outbreaks of JE [1]. In addition, JE immunization is difficult to organize in mountainous regions, with inadequate highways and people scattered over long distances, living away from urban areas. Accordingly, the incidence of JE in these southwestern regions remains higher than the national average. Thus, economic and social conditions are important factors for JE prevention and control in mainland China.

## Other Public Health Measures

The Chinese Ministry of Health has urged all levels of local government to conduct public outreach programs to improve knowledge regarding JE prevention, to launch campaigns for mosquito control during the summer breeding season, and to provide funding for JE vaccination of school-aged children [70]. In addition, the provincial Centers for Disease Control and Prevention (CDCs) have been instructed to establish JE laboratory testing systems and strengthen the capacity for laboratory diagnosis. The JE Laboratory Network in China was successfully set up based on the National JE Laboratory, which had been established by the Chinese Center for Disease Control and Prevention (China CDC). The missions of laboratories involved in the national network include laboratory testing of JE cases, vector surveillance, and epidemiological investigation of each confirmed case to provide basic information for local JE prevention and control. Staff from the JE Laboratory Network are trained in JE testing techniques through annual hands-on training programs provided by the National JE Laboratory. In addition, staff from the National JE Laboratory attend annual hands-on training workshops regarding JE lab testing organized by the World Health Organization (WHO). The central government has provided funding to establish an Internet-based, direct-reporting system to ensure that cases of 39 Notifiable Infectious Diseases, including JE, can be reported to the China CDC in a timely and accurate manner [63]. The China CDC is responsible for analyzing epidemiological data after receiving the reported data to provide evidence for the Ministry of Health to develop strategies for disease prevention and control. Furthermore, a special JE reporting system was established in 2006 in which the local CDCs developed electronic immunization records for every immunized child, thereby avoiding repeated immunization of the same child [1].

## Experiences and Challenges

Although these immunization programs have markedly reduced the incidence of JE in China in recent years, the continuing high incidence in the underdeveloped southwest region warrants further attention and requires strengthening existing JE immunization programs and comprehensive management. Moreover, a study indicated that some reported cases of JE included individuals that were subsequently confirmed to have been infected not by JEV but by other viruses, such as ECHO virus, coxsackie virus, herpes simplex virus, mumps virus, or cytomegalovirus, indicating the urgent need to improve the standards of laboratory testing [71]. In addition, Xinjiang, Tibet, and Qinghai are considered regions where JE is not prevalent, and no JE cases have been reported in these regions since 1951 [1], [2]. However, JEV was isolated from *C. tritaeniorhynchus* mosquitoes collected from Tibet for the first time in 2009 [72], [73], and antibody-screening results showed that neutralizing antibody to JEV was positive in local healthy people and domestic pigs. These observations suggest that Tibet may have become a new focus of JE. Thus, the detection and surveillance of JE cases should be strengthened in areas that were traditionally considered not to have JE cases, to provide a basis for the establishment of a local EPI.

Approximately 25%–30% of all JE cases are fatal, with irreversible neurological sequelae in up to 50% of the survivors [74]. This presents a huge burden on the family and society as a whole. Fortunately, however, JE has now become a cost-effective vaccine-preventable disease. The incidence of JE in some countries, such as Japan and Korea, has been reduced almost to the extent of eradication by immunization programs. Mainland China has now also controlled JE through immunization and the incidence appears to have been reduced to a low level [1], [2], [75], [76]. Therefore, in JE-endemic countries with the financial resources and public health infrastructure, it is recommended that JE vaccination should be included in their national health programs. Nevertheless, JEV has an endemic transmission cycle involving mosquitoes and birds. This natural cycle is also amplified in countries where intensive pig farming takes place. Consequently, total elimination of JE is virtually impossible, since this natural reservoir will not be impacted by a human vaccination program. Thus, for countries with relatively poor resources and public health infrastructures, immunization programs should target high JE prevalence regions. Simultaneously, local health authorities should provide advice on measures to avoid exposure to mosquitoes, and also develop land management programs to reduce the incidence of mosquitoes in the endemic areas. This could be achieved relatively cheaply through education programs on how to avoid high-risk areas, and also by recruiting and training local residents in larviciding procedures and management of water storage containers in inhabited areas. Such mosquito control and education measures have proved to be quite successful in countries such as Japan and Korea, and in some regions of China (as described in this manuscript).

**Key Learning Points**

- China has a long history of high prevalence of Japanese encephalitis, with thousands of cases reported annually and incidence rates often exceeding 15/100,000 historically.
- The introduction of vaccines, developed in China, combined with an intensive vaccination program initiated during the late 1970s, as well as other public health interventions, has dramatically decreased the incidence of JE.
- The provided valuable experience could be directly applicable to the control of vector-borne diseases around the world for the benefit of health authorities throughout Asia and, potentially, worldwide.

**Top Five Papers**

1. Campbell GL, Hills SL, Fischer M, Jacobson JA, Hoke CH, et al. (2011) Estimated global incidence of Japanese encephalitis: a systematic review. Bull World Health Organ 89: 766–774.
2. Zheng YY, Li MH, Wang HY, Liang GD (2012) Japanese encephalitis and Japanese encephalitis virus in mainland China. Rev Med Virol 22: 301–322.
3. Ding D, Kilgore PE, Clemens J, Liu W, Xiu ZY (2003) Cost–effectiveness of routine immunization to control Japanese encephalitis in Shanghai, China. Bull World Health Organ 81: 334–342.
4. Fischer M, Hills S, Staples E, Johnson B, Yaich M, et al. (2008) Japanese encephalitis prevention and control: advances, challenges, and new initiatives. In: Scheld WM, Hammer SM, Hughes JM, editors. Emerging infections 8. Washington: ASM Press. pp. 93–124.
5. Yin ZD, Asay GR, Zhang L, Li YX, Zuo SY, et al. (2012) An economic evaluation of the use of Japanese encephalitis vaccine in the expanded program of immunization of Guizhou province, China. Vaccine 30: 5569–5577.
